# Impact of diagnostic laparoscopy on resectability and treatment strategy in FIGO III–IV ovarian cancer

**DOI:** 10.1007/s00404-025-08173-6

**Published:** 2025-09-10

**Authors:** H. Endres, D. Dayan, F. Ebner, S. Huwer, D. Jakob, M. Medl, L. Yagcioglu, I. Juhasz-Boess, F. A. Taran, L. Jung

**Affiliations:** 1 Department of Obstetrics and Gynecology, University Medical Center Freiburg, Freiburg, Germany; 2 Department of Gynecology and Obstetrics, University Hospital Ulm, Ulm, Germany; 3 Department of Obstetrics and Gynecology, Faculty of Medicine, University Hospital Cologne, University of Cologne, Cologne, Germany

**Keywords:** Ovarian cancer, Diagnostic laparoscopy, Cytoreductive surgery, Neoadjuvant chemotherapy, Operability assessment, Surgical planning

## Abstract

**Objective:**

To investigate the clinical utility of diagnostic laparoscopy in guiding treatment strategy and surgical outcomes for patients with advanced-stage ovarian cancer, specifically regarding operability assessment and the likelihood of complete cytoreduction.

**Methods:**

This retrospective cohort study analyzed 183 patients with histologically confirmed International Federation of Gynecology and Obstetrics (FIGO) stage III–IV ovarian cancer treated with curative intent between January 2018 and December 2023 at a tertiary referral center. Patients were divided into two groups: those who underwent diagnostic laparoscopy prior to primary treatment (*n* = 80) and those managed without laparoscopy (*n* = 103). Laparoscopy was selectively employed when operability was uncertain. The primary outcome was the rate of complete macroscopic tumor resection. Secondary endpoints included intraoperative inoperability, neoadjuvant chemotherapy (NACT) rates, and surgical complexity. Statistical analyses included chi-square tests and predictive value calculations.

**Results:**

Complete macroscopic resection was achieved in 57.5% of patients in the laparoscopy group compared to 68.0% in the control group. Among FIGO III cases, complete resection was lower in the laparoscopy group (63.0% vs. 77.0%), while rates were similar for FIGO IV (53.8% vs. 54.8%). Diagnostic laparoscopy had a positive predictive value of 59% and was a statistically significant, albeit weak, predictor of operability (*p* = 0.003, phi = 0.13). Patients in the laparoscopy group were more frequently triaged to NACT (78.8% vs. 50.5%). Intraoperative inoperability was also higher (29% vs. 14%).

**Conclusion:**

Diagnostic laparoscopy influenced treatment strategy by increasing NACT use and reducing non-beneficial surgeries. Though it did not improve overall cytoreduction rates, it enabled personalized treatment planning, especially in patients with ambiguous resectability, thereby potentially lowering surgical morbidity.

## What does this study adds to the clinical work

**Table:** 

Diagnostic laparoscopy helps guide treatment selection in advanced ovarian cancer by identifying patients better suited for neoadjuvant chemotherapy and reducing unnecessary surgeries, even though it does not increase overall cytoreduction rates.	

## Introduction

Ovarian cancer is the most lethal gynecologic malignancy, with more than 70% of patients diagnosed at International Federation of Gynecology and Obstetrics (FIGO) stage III–IV, largely due to nonspecific early symptoms and the lack of effective screening tools [1, 2]. In this setting, the extent of cytoreductive surgery remains the most critical prognostic factor, with complete macroscopic tumor resection strongly associated with improved survival [3, 4]. Despite advances in imaging, accurate preoperative assessment of resectability remains difficult. Conventional modalities such as CT, MRI, and colonoscopy have limited sensitivity in detecting subtle yet decisive patterns of peritoneal disease, such as low-volume carcinomatosis, mesenteric root involvement, or diaphragmatic infiltration [5–8].

To address these limitations, diagnostic laparoscopy has been proposed as a tool that enables direct intra-abdominal assessment, allowing better evaluation of tumor spread and real-time histologic confirmation, particularly in cases where image-guided biopsies fail or remain inconclusive [9, 10]. Laparoscopy-based scoring systems, such as the Predictive Index Value (PIV) developed by Fagotti et al., have been introduced to quantify the probability of complete resection. A PIV score ≥ 8 is considered predictive of poor operability and can guide clinicians toward selecting neoadjuvant chemotherapy (NACT) followed by interval cytoreductive surgery (ICS) rather than proceeding with primary cytoreductive surgery (PCS) [11, 12]. This approach is supported by randomized trials including EORTC 55971 and CHORUS, which have shown that NACT followed by ICS is non-inferior to PCS in terms of overall survival, while potentially offering reduced perioperative morbidity [13, 14]. The SCORPION trial further reinforced this perspective by demonstrating fewer perioperative complications when NACT was applied in carefully selected patients [15]. Despite its clinical potential, the routine integration of diagnostic laparoscopy into ovarian cancer management remains a subject of ongoing debate. Current guidelines offer only cautious recommendations, primarily due to concerns regarding interobserver variability, inconsistent predictive accuracy across institutions, and the risk of overtreatment with NACT [1, 2, 16, 17]. Nevertheless, emerging evidence suggests that when used selectively, laparoscopy may improve cost-effectiveness by reducing the incidence of non-therapeutic high-risk surgeries [17]. Results from the TRUST trial, which investigates the optimal timing of surgical intervention, show no significant survival advantage of primary cytoreductive surgery (PCS) in non-frail patients. These findings further highlight the need for reliable preoperative assessment tools—such as diagnostic laparoscopy—to support effective treatment stratification [18].

At our institution, diagnostic laparoscopy is used selectively in patients with suspected inoperability or ambiguous resectability based on clinical and radiologic criteria. The aim of this study is to evaluate the real-world impact of this approach on treatment planning, surgical outcomes, and operability prediction in patients with FIGO stage III–IV ovarian cancer. By analyzing outcomes in patients with and without laparoscopy, we seek to clarify its clinical utility as a decision-making adjunct in a contemporary oncologic setting.

## Methods

This retrospective cohort study was conducted at the University Medical Center Freiburg and received ethics approval from the institutional review board (Reference: 24–1364-S1-retro). The study included 183 female patients, aged 18 years or older, who were diagnosed with histologically confirmed FIGO stage III or IV ovarian cancer. All patients underwent treatment with curative intent between January 2018 and December 2023, in accordance with institutional protocols aligned with international guidelines [1, 2].

Patients were allocated into two groups based on whether diagnostic laparoscopy was performed prior to the initiation of their primary treatment. The laparoscopy group consisted of 80 patients who underwent diagnostic laparoscopy to assess operability before the treatment strategy—either PCS or NACT and ICS—was determined. The control group comprised 103 patients who were managed without laparoscopy and were directly assigned to PCS or NACT and ICS based on clinical and radiologic evaluation alone. Importantly, diagnostic laparoscopy was not used prior to ICS, and its application was determined at the discretion of the treating physicians in cases of suspected inoperability or ambiguous resectability. Final treatment strategies were established through interdisciplinary tumor board discussions incorporating clinical, imaging, and, where applicable, laparoscopic findings. Chemotherapy was administered as six cycles of intravenous treatment, either following PCS or as three cycles before and three cycles after ICS.

Comprehensive data were collected for all patients, including demographic and clinical baseline characteristics such as age, body mass index (BMI), American Society of Anesthesiologists (ASA) physical status classification, preoperative serum creatinine, preoperative serum CA-125 levels, and FIGO tumor stage. Histological subtype, particularly the presence of high-grade serous carcinoma, was also documented. Treatment-related variables included whether patients received PCS or NACT and ICS. Surgical parameters evaluated included the frequency of colorectal and small bowel resections, intraoperative findings of inoperability, and presence or absence of tumor infiltration in the resected bowel. Surgical outcomes were measured by the rate of macroscopic complete tumor resection, stratified by FIGO stage (III vs. IV) and by type of surgery (PCS vs. ICS).

Statistical analyses were conducted using Microsoft Excel 2024 and SPSS Version 29.0. Descriptive statistics were used to summarize baseline and treatment characteristics. Categorical variables were compared between groups using chi-square tests or Fisher’s exact tests, as appropriate. The predictive value of diagnostic laparoscopy in forecasting complete macroscopic resection was assessed using sensitivity, specificity, and positive predictive value (PPV). A p-value of less than 0.05 was considered statistically significant.

## Results

**Fig. 1:**
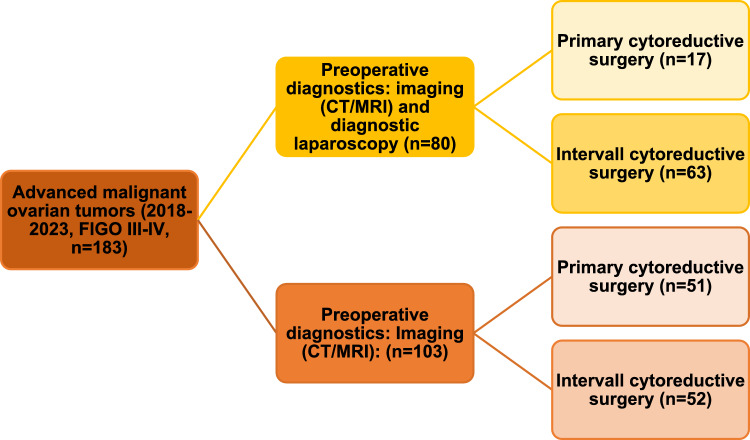
Patients with advanced ovarian tumors (FIGO III & IV) included in the study

A total of 183 patients with histologically confirmed FIGO stage III–IV ovarian cancer were included in the analysis. Of these, 80 patients underwent diagnostic laparoscopy prior to initial treatment planning, while 103 patients were managed without laparoscopy (Fig. 1).

The mean age was higher in the laparoscopy group compared to the control group (65.6 vs. 61.9 years; *p* = 0.04). No statistically significant differences were observed between the groups in terms of body mass index, ASA physical status classification, preoperative serum creatinine levels, or preoperative CA-125 levels. FIGO stage IV disease was present in 32.5% of the laparoscopy group and 40.8% of the control group. High-grade serous carcinoma was the most common histologic subtype in both groups, accounting for 65.0% of cases in the laparoscopy group and 60.2% in the control group. Neoadjuvant chemotherapy was administered to 78.8% of patients in the laparoscopy group and 50.5% in the control group (Table 1).

**Table 1:** Descriptive parameters of the study and control group

Patients with advanced ovarian carcinoma (FIGO III-IV)	Study group: diagnostic laparoscopy (*n* = 80)	Control group: no laparoscopy performed (*n* = 103)	t–test: *p*-value
Age (in years, mean; median)	mean: 65.6 median: 67	mean: 61.9; median: 62	0.04
BMI (mean; median)	mean: 24.7 median: 24.0	mean: 24.9 median: 24.5	0.81
ASA (mean; median)	mean: 2.6 median: 3	mean: 2.7 median: 3	0.15
Preoperative serum creatinin level (in mg/dL, mean; median)	mean: 0.79 median: 0.75	mean: 0.83 median: 0.78	0.33
Preoperative serum CA 125 level (in U/mL, mean; median)	mean: 534.6; median: 232.0	mean: 485.4; median: 128.0	0.76
Tumor Stage according to FIGO			
Figo III	67.5% (*n* = 54)	59.2% (*n* = 61)	
Figo IV	32.5% (*n* = 26)	40.8% (*n* = 42)	
Histology			
High grade serous ovarian carcinoma	65.0% (*n* = 52)	60.2% (*n* = 62)	
Neoadjuvant chemotherapy performed	78.8% (*n* = 63)	50.5% (*n* = 52)	

Primary cytoreductive surgery was performed in 21.3% of patients in the laparoscopy group and in 49.5% of the control group. Intraoperative inoperability during PCS occurred in 29.0% of the laparoscopy group and 14.0% of the control group. The rate of complete macroscopic tumor resection was 57.5% in the laparoscopy group and 68.0% in the control group. When stratified by FIGO stage, complete resection was achieved in 63.0% of FIGO III patients in the laparoscopy group and 77.0% in the control group. In patients with FIGO IV disease, complete resection was achieved in 53.8% of the laparoscopy group and 54.8% of the control group. Among patients who underwent PCS, complete macroscopic resection was achieved in 58.8% of the laparoscopy group and in 72.5% of the control group. In the subset of FIGO III patients undergoing PCS, complete resection was documented in 70.0% of the laparoscopy group and 81.6% of the control group. For FIGO IV patients undergoing PCS, complete resection was achieved in 57.1% of the laparoscopy group and 46.2% of the control group. Among patients undergoing ICS following NACT, complete resection was observed in 57.1% of the laparoscopy group and 63.5% of the control group. Within the FIGO III subgroup, complete resection after ICS was achieved in 61.4% of the laparoscopy group and 69.6% of the control group. In FIGO IV cases, complete resection was documented in 52.6% of the laparoscopy group and 58.6% of the control group. Colorectal resections were performed in 27.5% of the laparoscopy group and 35.9% of the control group. Colorectal shaving was documented in 1.3% of patients in the laparoscopy group and 4.9% in the control group. Small bowel resections were performed in 10.0% of the laparoscopy group and 15.5% of the control group. Intraoperative assessment found no bowel infiltration in 38.8% of laparoscopy group patients and 37.9% of control group patients for colorectal sites, and in 58.8% and 63.1%, respectively, for small bowel involvement. Diagnostic laparoscopy demonstrated a positive predictive value of 59% for achieving complete resection. Statistical analysis showed that laparoscopy was a significant, though weak, predictor of operability (chi-square = 8.815, *p* = 0.003; Fisher’s exact test *p* = 0.0053; phi = 0.13) (Table 2).

**Table 2:** Surgical outcome parameters of the study and control group, (Interval cytoreductive surgery (ICS); primary cytoreductive surgery (PCS))

Patients with advanced ovarian carcinoma (FIGO III-IV)	Study group: diagnostic laparoscopy (*n* = 80)	Control group: no laparoscopy performed (*n* = 103)
Colorectal resection/shaving		
Resection	27.5% (*n* = 22)	35.9% (*n* = 37)
Shaving	1.3% (*n* = 1)	4.9% (*n* = 5)
Inoperability	32.5% (*n* = 26)	21.4% (*n* = 22)
No infestation (appendectomy for mucinous carcinoma if necessary)	38.8% (*n* = 31)	37.9% (*n* = 39)
Small bowel resection		
Resection	10.0% (*n* = 8)	15.5% (*n* = 16)
Shaving	1.3% (*n* = 1)	0.0% (*n* = 0)
Inoperability	30.0% (*n* = 24)	21.4% (*n* = 22)
No infestation	58.8% (*n* = 47)	63.1% (*n* = 65)
Postoperative tumor residue		
Macroscopic complete resection	57.5% (*n* = 46)	68.0% (*n* = 70)
Figo III macroscopic complete resection: *(study group: n* = *54; control group: n* = *61)*	63.0% (*n* = 34)	77.0% (*n* = 47)
Figo IV macroscopic complete resection: *(study group: n* = *26; control group: n* = *42)*	53.8% (*n* = 14)	54.8% (*n* = 23)
Primary cytoreductive surgery: tumor residue	Study group: diagnostic laparoscopy and PCS (*n* = 17)	Control group: no laparoscopy performed and PCS (*n* = 51)
Macroscopic complete resection	58.8% (*n* = 10)	72.5% (*n* = 37)
Figo III macroscopic complete resection: *(study group: n* = *10; control group: n* = *38)*	70.0% (*n* = 7)	81.6% (*n* = 31)
Figo IV macroscopic complete resection:* (study group: n* = *7; control group: n* = *13)*	57.1% (*n* = 4)	46.2% (*n* = 6)
Intervall cytoreductive surgery: tumor residue	Study group: diagnostic laparoscopy and ICS (*n* = 63)	Control group: no laparoscopy performed and ICS (*n* = 52)
Macroscopic complete resection	57.1% (*n* = 36)	63.5% (*n* = 33)
Figo III macroscopic complete resection: *(study group: n* = *44; control group: n* = *23)*	61.4% (*n* = 27)	69.6% (*n* = 16)
Figo IV macroscopic complete resection: *(study group: n* = *19; control group: n* = *29)*	52.6% (*n* = 10)	58.6% (*n* = 17)

## Discussion

This study evaluated the impact of diagnostic laparoscopy on surgical decision-making and operative outcomes in patients with advanced ovarian cancer. While the overall rate of complete macroscopic cytoreduction was lower in the laparoscopy group (57.5%) compared to the control group (68.0%), these results must be interpreted within the context of patient selection. Notably, patients in the laparoscopy group were more frequently suspected of having unresectable disease based on clinical and imaging features, and laparoscopy was purposefully used to further assess operability. This reflects a targeted application of laparoscopy in patients with unclear or borderline resectability.

The higher rate of NACT administration in the laparoscopy group (78.8% vs. 50.5%) underscores the role of laparoscopy in influencing treatment pathways. This finding is consistent with prior studies demonstrating that laparoscopy may direct patients with high tumor burden or those deemed poor surgical candidates toward NACT, thereby potentially reducing the frequency of non-beneficial laparotomies and associated morbidity. Furthermore, laparoscopy may help to avoid the morbidity associated with aggressive primary cytoreductive surgery in patients unlikely to achieve complete resection, aligning with the morbidity reduction strategy described in the SCORPION trial [15]. Although complete resection rates were not improved by laparoscopy and were, in fact, slightly lower, the increased inoperability rate (29% vs. 14%) in the laparoscopy group likely reflects a cohort with more extensive or complex disease rather than a failure of surgical execution. Importantly, the observed outcomes support the notion that diagnostic laparoscopy appropriately identified patients for whom immediate surgery would not have been beneficial. This finding aligns with the intended purpose of staging laparoscopy: not to directly increase resection rates across all cases, but rather to improve triage and reduce futile or overly morbid procedures. In addition, laparoscopy allowed for direct histologic sampling when needed and provided real-time evaluation of intra-abdominal disease spread, such as mesenteric or diaphragmatic involvement—factors often underestimated by imaging alone [5–10, 19]. This facilitated early initiation of appropriate treatment without the delays associated with inconclusive imaging or less accessible biopsy approaches.

While the predictive value of laparoscopy in identifying operable cases was modest (PPV 59%), it was statistically significant (*p* = 0.003) and supports its use in conjunction with radiologic and clinical evaluation. The effect size was small (phi = 0.13), but this may reflect real-world variability in laparoscopy scoring systems or interobserver differences in disease interpretation. Importantly, the relatively lower resection rates observed in the laparoscopy group should not be misinterpreted as a negative outcome; rather, they demonstrate effective risk stratification and a rational application of NACT in patients less likely to benefit from PCS.

Notably, differences in bowel resections—particularly the lower rates of small bowel and colorectal resections in the laparoscopy group—further support the hypothesis that laparoscopy may help avoid unnecessary high-complexity surgery in patients ultimately not suited for it. This supports previous reports suggesting that staging laparoscopy may help optimize surgical burden in individual patients [11, 12, 16].

This study’s strengths include a relatively large cohort and the incorporation of real-world data from a tertiary referral center, reflecting contemporary treatment decisions in advanced ovarian cancer. However, limitations include its retrospective design and potential selection bias, as patients selected for laparoscopy may have had different baseline characteristics or disease patterns not fully accounted for. In addition, standardized laparoscopy scoring systems such as the Predictive Index Value (PIV) were not uniformly recorded, limiting further stratified analysis.

## Conclusion

In conclusion, diagnostic laparoscopy in advanced ovarian cancer did not improve overall cytoreduction rates but served a critical role in preoperative decision-making, particularly when complete resection was uncertain. Patients selected for laparoscopy often had a higher risk of inoperability, and the procedure helped identify those better suited for neoadjuvant chemotherapy, potentially avoiding non-therapeutic laparotomies and reducing surgical morbidity. Laparoscopy should be considered a valuable adjunct in treatment stratification, especially in complex or ambiguous cases. Prospective, randomized studies are needed to establish standardized criteria for selecting complex or ambiguous cases in which laparoscopic staging is beneficial.

## Data Availability

No datasets were generated or analyzed during the current study.

## References

[1] Leitlinienprogramm Onkologie (Deutsche Krebsgesellschaft, Deutsche Krebshilfe, AWMF): S3-Leitlinie Diagnostik, Therapie und Nachsorge maligner Ovarialtumoren, Langversion 6.0, 2024, AWMF-Registernummer: 032–035OL https://www.leitlinienprogramm-onkologie.de/leitlinien/ovarialkarzinom/; Zugriff am [19.02.2025]j

[2] Colombo N, Sessa C, du Bois A et al (2019) ESMO–ESGO consensus conference recommendations on ovarian cancer: pathology and molecular biology, early and advanced stages, borderline tumours and recurrent disease. Ann Oncol 30:672–705. 10.1093/annonc/mdz062 31046081 10.1093/annonc/mdz062

[3] du Bois A, Reuss A, Pujade-Lauraine E et al (2009) Role of surgical outcome as prognostic factor in advanced epithelial ovarian cancer: a combined exploratory analysis of 3 prospectively randomized phase 3 multicenter trials. Cancer 115:1234–1244. 10.1002/cncr.24149 19189349 10.1002/cncr.24149

[4] Jakob D, Schmoor C, Reuten R et al (2023) Characteristics, treatment patterns and survival of International federation of gynecology and obstetrics stage IV epithelial ovarian cancer—a population-based study. Cancers (Basel) 15:5676. 10.3390/cancers15235676 38067378 10.3390/cancers15235676 PMC10705193

[5] Gómez-Hidalgo NR, Martinez-Cannon BA, Nick AM et al (2015) Predictors of optimal cytoreduction in patients with newly diagnosed advanced-stage epithelial ovarian cancer: time to incorporate laparoscopic assessment into the standard of care. Gynecol Oncol 137:553–558. 10.1016/j.ygyno.2015.03.049 25827290 10.1016/j.ygyno.2015.03.049 PMC4825172

[6] Endres H, Daneehl D, Fichtner-Feigl S et al (2025) Preoperative colonoscopy in ovarian cancer: impact on surgical planning and outcomes: results from a retrospective, single-center study. Arch Gynecol Obstet. 10.1007/s00404-025-08086-4 40517190 10.1007/s00404-025-08086-4 PMC12374851

[7] Rutten MJ, van Meurs HS, van de Vrie R et al (2017) Laparoscopy to predict the result of primary cytoreductive surgery in patients with advanced ovarian cancer: a randomized controlled trial. J Clin Oncol 35:613–621. 10.1200/JCO.2016.69.2962 28029317 10.1200/JCO.2016.69.2962

[8] Axtell AE, Lee MH, Bristow RE et al (2007) Multi-institutional reciprocal validation study of computed tomography predictors of suboptimal primary cytoreduction in patients with advanced ovarian cancer. J Clin Oncol 25:384–389. 10.1200/JCO.2006.07.7800 17264334 10.1200/JCO.2006.07.7800

[9] Griffin N, Grant LA, Freeman SJ et al (2009) Image-guided biopsy in patients with suspected ovarian carcinoma: a safe and effective technique? Eur Radiol 19:230–235. 10.1007/s00330-008-1121-8 18704437 10.1007/s00330-008-1121-8

[10] Souza FF, Mortelé KJ, Cibas ES et al (2009) Predictive value of percutaneous imaging-guided biopsy of peritoneal and omental masses: results in 111 patients. AJR Am J Roentgenol 192:131–136. 10.2214/AJR.08.1283 19098191 10.2214/AJR.08.1283

[11] Fagotti A, Ferrandina G, Fanfani F et al (2008) Prospective validation of a laparoscopic predictive model for optimal cytoreduction in advanced ovarian carcinoma. Am J Obstet Gynecol 199:642.e1-642.e6. 10.1016/j.ajog.2008.06.052 18801470 10.1016/j.ajog.2008.06.052

[12] Fagotti A, Ferrandina G, Fanfani F et al (2006) A laparoscopy-based score to predict surgical outcome in patients with advanced ovarian carcinoma: a pilot study. Ann Surg Oncol 13:1156–1161. 10.1245/ASO.2006.08.021 16791447 10.1245/ASO.2006.08.021

[13] Vergote I, Coens C, Nankivell M et al (2018) Neoadjuvant chemotherapy versus debulking surgery in advanced tubo-ovarian cancers: pooled analysis of individual patient data from the EORTC 55971 and CHORUS trials. Lancet Oncol 19:1680–1687. 10.1016/S1470-2045(18)30566-7 30413383 10.1016/S1470-2045(18)30566-7

[14] Kehoe S, Hook J, Nankivell M et al (2015) Primary chemotherapy versus primary surgery for newly diagnosed advanced ovarian cancer (CHORUS): an open-label, randomised, controlled, non-inferiority trial. Lancet 386:249–257. 10.1016/S0140-6736(14)62223-6 26002111 10.1016/S0140-6736(14)62223-6

[15] Fagotti A, Ferrandina MG, Vizzielli G et al (2020) Randomized trial of primary debulking surgery versus neoadjuvant chemotherapy for advanced epithelial ovarian cancer (SCORPION-NCT01461850). Int J Gynecol Cancer 30:1657–1664. 10.1136/ijgc-2020-001640 33028623 10.1136/ijgc-2020-001640

[16] van de Vrie R, Rutten MJ, Asseler JD et al (2019) Laparoscopy for diagnosing resectability of disease in women with advanced ovarian cancer. Cochrane Database Syst Rev 3:CD009786. 10.1002/14651858.CD009786.pub3 30907434 10.1002/14651858.CD009786.pub3 PMC6432174

[17] van de Vrie R, van Meurs HS, Rutten MJ et al (2017) Cost-effectiveness of laparoscopy as diagnostic tool before primary cytoreductive surgery in ovarian cancer. Gynecol Oncol 146:449–456. 10.1016/j.ygyno.2017.06.019 28645428 10.1016/j.ygyno.2017.06.019

[18] Mahner S, Heitz F, Salehi S et al (2025) TRUST: trial of radical upfront surgical therapy in advanced ovarian cancer (ENGOT ov33/AGO-OVAR OP7). J Clin Oncol. 10.1200/JCO.2025.43.17_suppl.LBA550039353164

[19] Hewitt M, Anderson K, Hall G et al (2007) Women with peritoneal carcinomatosis of unknown origin: efficacy of image-guided biopsy to determine site-specific diagnosis. BJOG 114:46–50. 10.1111/j.1471-0528.2006.01176.x 17233859 10.1111/j.1471-0528.2006.01176.x

